# Genomic modifiers of malignant and neurodevelopmental phenotypes in individuals with *PTEN* hamartoma tumor syndrome

**DOI:** 10.1038/s41525-026-00556-1

**Published:** 2026-03-17

**Authors:** Lamis Yehia, Lin Li, Gideon Idumah, Thomas W. Frazier, Vladimir Makarov, Aritra Bose, Laxmi Parida, Antonio Hardan, Julian A. Martinez-Agosto, David M. Ritter, Mustafa Sahin, Charis Eng, Ying Ni

**Affiliations:** 1 Genomic Medicine Institute, Cleveland Clinic Research, Cleveland Clinic, Cleveland, OH USA; 2 Epilepsy Center, Neurological Institute, Cleveland Clinic, Cleveland, OH USA; 3 Cancer Sciences, Cleveland Clinic Research, Cleveland Clinic, Cleveland, OH USA; 4 Autism Speaks, Cleveland, OH USA; 5 Department of Psychology, John Carroll University, University Heights, OH USA; 6 Departments of Pediatrics and Psychiatry, SUNY Upstate Medical University, Syracuse, NY USA; 7 IBM Research, Yorktown Heights, NY USA; 8 Psychiatry and Behavioral Sciences, Stanford University Medical Center, Stanford, CA USA; 9 Departments of Pediatrics and Human Genetics, David Geffen School of Medicine at UCLA, Los Angeles, CA USA; 10 Division of Neurology, Cincinnati Children’s Hospital Medical Center; Department of Pediatrics, University of Cincinnati College of Medicine, Cincinnati, OH USA; 11 Department of Neurology, Boston Children’s Hospital, Harvard Medical School, Boston, MA USA; 12 F.M. Kirby Neurobiology Center, Boston Children’s Hospital, Harvard Medical School, Boston, MA USA; 13 Center for Personalized Genetic Healthcare, Community Care, Cleveland Clinic, Cleveland, OH USA; 14 Taussig Cancer Institute, Cleveland Clinic, Cleveland, OH USA; 15 Department of Genetics and Genome Sciences, Case Western Reserve University School of Medicine, Cleveland, OH USA; 16 Germline High Risk Cancer Focus Group, CASE Comprehensive Cancer Center, Case Western Reserve University, Cleveland, OH USA; 17 School of Medicine, Case Western Reserve University, Cleveland, OH USA; 18 Epilepsy Center Department of Psychiatry & Psychology Neurological Institute Cleveland Clinic, Cleveland, OH USA; 19 Department of Developmental and Behavioral Pediatrics, Cincinnati Children’s Hospital Medical Center, Cincinnati, OH USA; 20 UCLA Semel Institute for Neuroscience & Human Behavior, David Geffen School of Medicine at UCLA, Los Angeles, CA USA; 21 Department of Developmental Medicine, Boston Children’s Hospital, Boston, MA USA; 22 Center for General Neurology, Cleveland Clinic, Cleveland, OH USA; 23 Department of Psychiatry, Stanford University, Stanford, CA USA

**Keywords:** Cancer, Genetics, Oncology

## Abstract

PTEN hamartoma tumor syndrome (PHTS), caused by germline *PTEN* variants, exhibits marked phenotypic heterogeneity, most notably cancer, neurodevelopmental disorders (NDD), or both. The basis for this divergence, even among carriers of identical *PTEN* variants, remains poorly defined. We performed whole-genome sequencing of 599 individuals with PHTS and family members, complemented by analyses of *PTEN* variant carriers from the All of Us Research Program. Analyses included both targeted evaluation of genes previously implicated in cancer and NDD and agnostic genome-wide single-variant and rare-variant burden testing. The analytic cohort comprised 543 PHTS probands, including individuals with NDD (*n* = 171), cancer (*n* = 221), both phenotypes (*n* = 21), or neither (*n* = 130) at the time of enrollment. Pathogenic or likely pathogenic variants in cancer-associated genes were identified in 37 (6.8%), most frequently in *MITF*, *DICER1*, and *BRCA2*, while 43 (7.9%) harbored variants in NDD-related genes, including *DHCR7*, *POLG*, and *ARSA*. Such secondary variants were less common in *PTEN* variant carriers in All of Us. Genome-wide analyses identified candidate modifier loci functionally linked to PTEN, including in *ZNF713*, *TPTE2P1*, and *PDPK1*. These findings demonstrate that PHTS phenotypes are shaped by complex gene–gene interactions beyond *PTEN* alone, informing mechanisms underlying the cancer–NDD dichotomy and advancing precision risk stratification.

## Introduction

Integrating human phenotypic data with “multi-omics” data, has emerged as a blueprint for precision medicine^1^. As such, the triumph of utilizing genetics in precision medicine is the ability to stratify individuals at very high risk of disease, with the ability to enact specific gene-informed medical management^2,3^. We have been on a mission of implementing this integrative approach for classic Mendelian disorders like PTEN hamartoma tumor syndrome (PHTS), starting from deeply phenotyped human model organisms, to identify physiologically relevant and clinically meaningful measures of disease risk. PHTS is diagnosed upon the identification of a pathogenic or likely pathogenic heterozygous germline *PTEN* mutation on molecular genetic testing, regardless of the phenotype.

The extensive phenotypic heterogeneity amongst individuals with PHTS represents a profound clinical challenge. Notwithstanding elevated lifetime risks of multiple cancer types^4–6^, *PTEN* germline mutations are also among the most common monogenic causes of neurodevelopmental disorders (NDD) including autism spectrum disorder (ASD) and intellectual disability (ID)^7–9^. Why one gene contributes to such disparate clinical manifestations, i.e., cancer versus NDD, including in patients with identical germline *PTEN* variants, remains poorly understood. This *PTEN*-related cancer-NDD phenotypic dichotomy and inability to preemptively predict disease outcomes pose a challenge for more timely and precise clinical management. However, based on this broad clinical spectrum and single genetic etiology (namely, *PTEN*), PHTS serves as a powerful model to identify modifiers of variable heritable disease risk at the individual level.

Accordingly, we started to reveal PHTS manifestations not as single gene-driven, but rather complex gene-modifier interactions, which could modulate the risk of disease development, tumor type and behavior, as well as phenotypic burden^7,10^. As proof-of-principle, we showed that genome-wide copy number variations (CNVs), mitochondrial genome (mtDNA) copy number and variant burden, as well as germline homozygosity burden act as genomic modifiers of cancer versus NDD risk in individuals with germline *PTEN* variants^11–13^. We also showed that metabolomic factors may act as modifiers of NDD versus cancer in PHTS^14,15^. However, these modifiers only occur in a subset of individuals with PHTS, suggesting the existence of yet to be identified modifiers. Thus, we sought to address this hypothesis using a comprehensive integrative genome sequencing approach in a series of individuals with PHTS.

## Results

### Patient characteristics and germline *PTEN* variant spectrum

At baseline, we genome sequenced DNA from 599 participants with PHTS and a subset of family members (median [IQR] age at consent, 35 [10–50] years; 330 female [55%] and 269 male [45%] participants) (Table 1). Of the 599 research participants, 259 (43%) received cancer diagnoses, with 21 of those also having neurodevelopmental disorders (NDD). Of the 340 patients without cancer, 181 (53%) received diagnoses of NDD, whereas 159 (27%) did not have cancer nor NDD at the time of consent (median [IQR] age at consent, 35 [15–47] years; 76 female [48%] and 83 male [52%] participants). This series also included 20 *PTEN* wildtype family members, regardless of cancer and NDD phenotypes. For the remaining 579 participants with germline *PTEN* variants, we classified the *PTEN* variants into tier 1 (481 variants [83%]) including all pathogenic (P) and likely pathogenic (LP) variants, and tier 2 (98 variants [17%]) including variants of uncertain significance (VUS, 79 [80.6%]), those with conflicting interpretation of pathogenicity including at least one P, LP, or VUS designation (11 [11.2%]), and a minority with likely benign variants (8 [8.2%]). After performing sample-level quality control, ancestral stratification, and genetic relatedness analysis, the analytic sample consisted of 543 probands with PHTS (median [IQR] age at consent, 34 [10–50] years; 293 female [54%] and 250 male [46%] participants). Phenotypic groups consisted of 171 patients with NDD, 221 patients with cancer, a subset of 21 patients with both NDD and cancer, and 130 patients without NDD or cancer at the time of consent (Table 1).

**Table 1:** Clinical phenotypic and genotypic characteristics of study participants

Clinical Characteristics	All Participants *N* = 599	Analytic Series *N* = 543
*Biological sex*		
Female	330 (55.1%)	293 (54.0%)
Male	269 (44.9%)	250 (46.0%)
*Median age at consent (IQR)*	35 (10-50)	34 (10-50)
*PHTS phenotype classifications*		
Cancer	238 (39.7%)	221 (40.7%)
Neurodevelopmental disorders (NDD)^a^	181 (30.2%)	171 (31.5%)
Cancer and NDD	21 (3.5%)	21 (3.9%)
No Cancer and no NDD	159 (26.5%)	130 (23.9%)
*Germline PTEN variant classifications*^b^		
Tier 1	485 (81.0%)	456 (84.0%)
Tier 2	94 (15.7%)	87 (16.0%)
Wildtype	20 (3.3%)	0 (0.0%)

*IQR* interquartile range, *PHTS* *PTEN* hamartoma tumor syndrome.

^a^Includes autism spectrum disorder (ASD), developmental delay (DD), and/or intellectual disability (ID).

^b^Tier 1 includes pathogenic and/or likely pathogenic *PTEN* variants; Tier 2 includes all other variants.

### Variants in known cancer and NDD predisposing genes

**Fig. 1:**
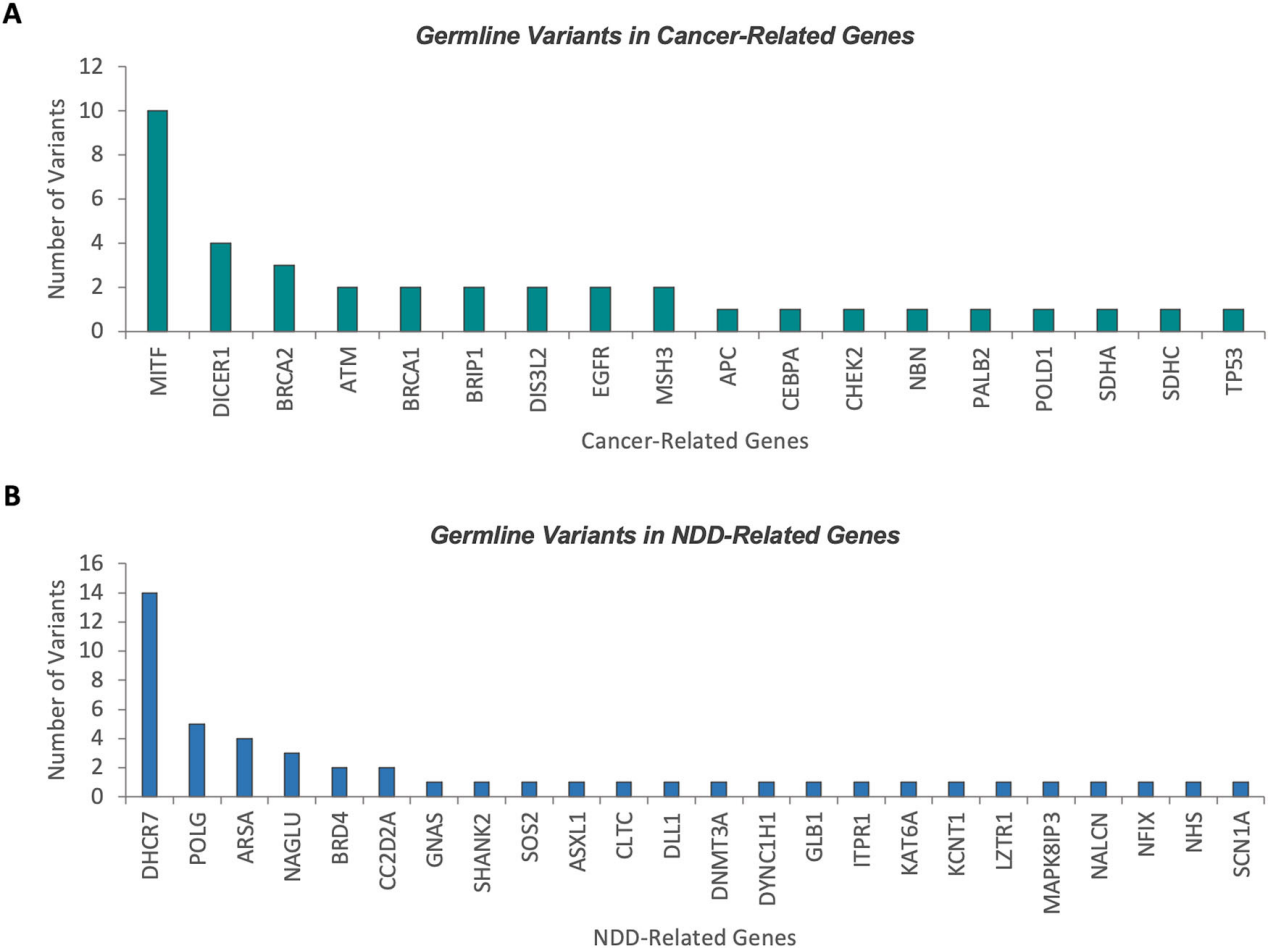
Germline variants in known cancer and NDD related genes. A subset of individuals with germline *PTEN* variants were found to harbor other germline variants in known cancer-related genes (**A**) and NDD-related genes (**B**). Variants are depicted according to descending order of prevalence. NDD, neurodevelopmental disorders.

We utilized the sequencing data to extract variants in genes known to be associated with cancer and NDD, irrespective of PHTS. The premise for this data analysis is to test the hypothesis that patients with PHTS have cancer or NDD/ASD because they have other germline variants, on top of the underlying germline *PTEN* variants, in genes known to be associated with either phenotype. Cancer-related genes covered 85 genes typically tested clinically in multi-gene panels, including actionable genes as defined by the ACMG (Supplementary Data 1). Overall, we identified 37/543 (6.8%) individuals with PHTS and germline variants in other cancer-related genes including *MITF* (*n* = 10), *DICER1* (*n* = 4), *BRCA2* (*n* = 3), and others (Fig. 1A). One individual (CCF02111-02-001) had germline variants in two cancer-related genes other than *PTEN*, namely *NBN* and *MITF* (Supplementary Data 3). Additionally, among carriers of both *PTEN* and additional cancer related gene variants, 29/37 (78.4%) have tier 1 (P/LP) germline *PTEN* variants. Of the 55 participants with germline *PTEN* variants from the All of Us Research Program dataset, none carried germline variants in other known cancer predisposition genes.

Genes associated with NDD/ASD covered three independent clinically tested panels, whose overlap results in 331 unique genes (Supplementary Data 2). We identified 43/543 (7.9%) patients with germline variants in NDD/ASD-associated genes (Supplementary Data 4). These variants occurred in 24 genes, the most prevalently mutated including *DHCR7* (*n* = 14), *POLG* (*n* = 5), *ARSA* (*n* = 4), and *NAGLU* (*n* = 3) (Fig. 1B). Of the 43 variant carriers, 15 (35%) presented with NDD/ASD-associated phenotypes. Additionally, among carriers of both *PTEN* and additional NDD related gene variants, 34/43 (79.1%) have tier 1 (P/LP) germline *PTEN* variants. Relatedly, of the 55 participants with germline *PTEN* variants from the All of Us Research Program dataset, we identified 2 (3.6%) participants with germline P/LP NDD-related variants, including one with a nonsense *DNM1L* variant (NM_001278463: c.28 A > T, p.K10X), and another with a splicing *DNMT3A* variant (NM_022552: c.1014+1 G > C).

### Analysis of genome-wide common variants

**Fig. 2:**
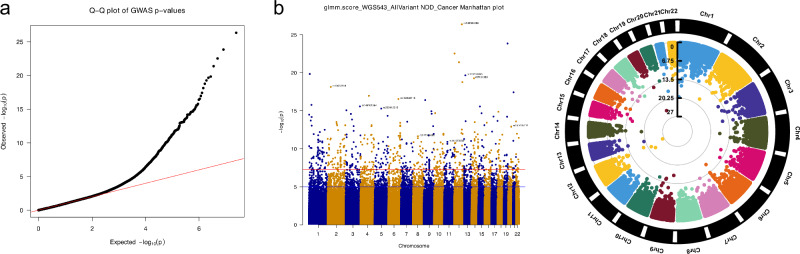
Analysis of genome-wide common variants. **a** Q-Q plot and (**b**) Manhattan plots of the NDD versus Cancer analysis in PHTS with WGS extracted single SNPs. The circular Manhattan plot depicts the common variants (MAF > 0.01) by chromosome (black and white bars at the circumference), and *p*-values depicted as concentric circles (the deeper the variants, the lower is the *p*-value). Suggestive significance threshold (black line) = 1 × 10^−5^; genome-wide significance threshold (red line) = 5 × 10^-8^.

Because known cancer and NDD associated gene variants only occur in a subset of individuals with PHTS and these phenotypes, we next performed an agnostic genome-wide approach to identify candidate genomic modifiers. For this analysis, we performed single variant tests covering common variants (minor allele frequency or MAF between 0.01 and 0.5) using the Generalized Mixed Model Association Test (GMMAT) analysis^16^. Age at consent, biological sex, and *PTEN* variant tier were used as covariates in the GMMAT model. For single common variant analysis, we used variants with 0.01 < MAF < 0.5, including a total of 12,476,111 variants, encompassing both coding and non-coding variants. Focusing on individuals with PHTS and NDD versus those with cancer, we identified 622,149 common variants with *P* value < 0.05, of which 748 variants have a *P* < 5 × 10^−8^ (Fig. 2 and Supplementary Data 5).

### Gene-based test of rare variants

**Fig. 3:**
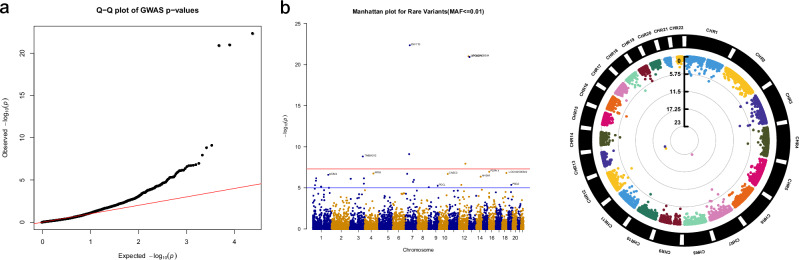
Gene-based test of rare variants. **a** Q-Q plot and (**b**) Manhattan plots of the NDD versus Cancer analysis in PHTS with WGS extracted rare variants (MAF ≤ 0.01). The circular Manhattan plot depicts the rare variants (MAF ≤ 0.01) by chromosome (black and white bars at the circumference), and p-values depicted as concentric circles (the deeper the variants, the lower is the *p*-value). Suggestive significance threshold (black line) = 1 × 10^−5^; genome-wide significance threshold (red line) = 5 × 10^-8^.

We implemented the GMMAT package to test the gene specific rare variant burden between PHTS individuals with NDD versus PHTS individuals with cancer, and to prioritize candidates by phenotypic relevance. This analysis included rare variants (MAF≤0.01), and covariates in the model included age at consent, biological sex, and *PTEN* variant tier. We identified multiple candidate modifier variants that are distinctly represented in PHTS patients based on NDD versus cancer phenotypes (Fig. 3). Importantly, we identified multiple candidate modifier genes in individuals with PHTS with either NDD or cancer phenotypes that are relevant to these phenotypes of interest and/or that crosstalk with the PTEN signaling pathway (Table 2).

**Table 2:** Top 10 significant candidate genes with rare variants

Gene	Description^a^	Chr	*N* variants	S.Pval	O.Pval	B.Pval	B.Effect_Size	B.SE	Enrichment
*ZNF713*	Zinc finger protein 713	7	2	2.91E-23	4.77E-23	5.96E-24	2.509	0.249	Cancer vs NDD
*LOC100128554*	Long intergenic non-protein coding RNA 2347	12	6	1.49E-22	1.04E-21	1.30E-22	0.719	0.073	Cancer vs NDD
*TPTE2P1*	TPTE2 pseudogene 1	13	1	1.25E-21	1.25E-21	1.25E-21	4.513	0.472	Cancer vs NDD
*HUS1*	HUS1 checkpoint clamp component	7	1	7.94E-10	7.94E-10	7.94E-10	1.784	0.290	Cancer vs NDD
*TMEM212*	Transmembrane protein 212	3	1	1.53E-09	1.53E-09	1.53E-09	1.661	0.275	Cancer vs NDD
*TSPAN19*	Tetraspanin 19	12	1	1.16E-08	1.16E-08	1.16E-08	1.578	0.277	Cancer vs NDD
*PDPK1*	3-phosphoinositide dependent protein kinase 1	16	1	1.11E-07	1.11E-07	1.11E-07	1.391	0.262	Cancer vs NDD
*LOC100505549*	ATP8B1 antisense RNA 1	18	1	1.56E-07	1.56E-07	1.56E-07	1.380	0.263	Cancer vs NDD
*ARSJ*	Arylsulfatase family member J	4	2	1.37E-07	1.84E-07	1.64E-05	0.481	0.112	Cancer vs NDD
*CDCA7L*	Cell division cycle associated 7 like	7	9	3.18E-06	1.99E-07	8.16E-08	0.199	0.037	Cancer vs NDD

*N* number, *S.Pval* SKAT *P* value, *O.Pval* SKAT-O *P* value, *B.Pval* Burden test *P* value, *B.Effect_Size* Burden test effect size, *B.SE* Burden test standard error.

^a^Descriptions are derived from Human Genome Organization (HUGO) Gene Nomenclature Committee (HGNC) database (https://www.genenames.org/). Information for uncharacterized genes were extracted from the National Center for Biotechnology Information (NCBI, https://www.ncbi.nlm.nih.gov/gene/).

## Discussion

Although it is well-established that PTEN plays a fundamental role in carcinogenesis and neurodevelopment^7^, determinants of phenotype manifestations in individuals with germline *PTEN* variants remain elusive. This observation is especially pertinent amongst individuals with identical *PTEN* variants and disparate phenotypes, including within the same family. In recent works, we focused on the hypothesis that modifiers can modulate phenotypic manifestations, with a focus on cancer and NDD as the most common and well-defined phenotypes in PHTS^17^. These data indicated that a complex gene-environment interaction, including microenvironmental factors such as metabolites, is likely to contribute to phenotype development across the lifespan^14,15^. Here, we investigated genome-wide modifiers first using a targeted approach, and then an agnostic discovery approach.

Through the targeted approach, we identified 37/543 (6.8%) individuals with PHTS and germline variants in other known cancer-related genes. Interestingly, 29/37 (78.4%) of carriers of these germline variants had tier 1 (P/LP) germline *PTEN* variants. Prior to the current systematic study, clinical case reports by our team and others have previously shown that germline *PTEN* variants occurred in combination with variants in *APC*, *SDHC*, and *TP53*, with concordant clinical manifestations in all three cases^18–20^. In this study, we identified concordant gene-phenotype associations in variant carriers, with 19/37 (51%) of carriers reported to have a cancer diagnosis. However, 11 of the 18 variant carriers without cancer were 18 years of age or younger. Only one of the cancer-related variant carriers was reported to have pediatric-onset cancer, namely thyroid cancer at age 10 years. The co-occurrence of germline variants in two or more cancer predisposition genes in the same individual forms the basis of multilocus inherited neoplasia alleles syndrome (MINAS), which becomes especially pertinent in patients with unusual presentations of inherited cancer predisposition syndromes^21,22^. In these cases, genotype-phenotype correlations become complicated, given the rarity of the syndromes and association of multiple genes with the same cancer phenotypes.

It is worth noting that germline variants in *MITF* c.952 G > A, p.E318K occurred in 10 individuals with PHTS. *MITF* encodes melanocyte inducing transcription factor and has been found to function as a melanoma oncogene, and an activator of hypoxia inducible factor 1 subunit alpha (HIF1A), which is also targeted by kidney cancer susceptibility genes^23^. Importantly, both melanoma and renal cell carcinoma (RCC) are PHTS component cancers^6^. The *MITF* E318K substitution results in a SUMOylation defective variant that enhances transcriptional activity, particularly on target genes involved in cell growth, proliferation, migration, and hypoxia responses^24,25^. Accordingly, studies have shown that *MITF* p.E318K carriers harbor an increased risk of developing melanoma, RCC, or co-occurrence of both cancers^24,25^. In our series, of the 6 adults harboring the *MITF* variant, one carrier had RCC at age 34, and another had melanoma at age 60. While studies have not identified increased risk for other malignancies in p.E318K carriers alone^26^, the most predominant cancer reported in PHTS *MITF* p.E318K carriers was that of the thyroid, reported in 4 of 6 adult carriers.

We identified 43/543 (7.9%) patients with germline variants in NDD/ASD-associated genes. Two illustrative examples of concordant genotype-phenotype associations include *DLL1* and *SHANK2* variants. *DLL1* c.1525 C > T, p.R509X occurred in a 19-year-old male with *PTEN* c.586_587insT, p.H196Lfs*6 variant presenting with dysmorphic features, epilepsy, global developmental delay, intellectual disability, and normal head size. Heterozygous variants in *DLL1* are associated with neurodevelopmental disorder with nonspecific brain abnormalities and with or without seizures (OMIM 618709)^27^. Relatedly, we identified a *SHANK2* variant in a 3-year-old male with *PTEN* c.176 C > G, p.S59X variant, presenting with macrocephaly, ASD, global developmental delay, dysmyelinating leukodystrophy, and congenital urinary tract anomalies. While it is intriguing to hypothesize that carriers with germline *PTEN* variants and additional NDD-related genes may have more severe neurodevelopmental manifestations, the current data are not sufficient to investigate this question. Importantly, we posit that the prevalence of such germline variants in the All of Us dataset is lower than that from our PTEN Multidisciplinary Clinic series (3.6% vs 7.9%), likely owing to referral bias and the enrichment of our dataset with children, compared to adult participants from All of Us. Relatedly, the AoU cohort lacked detailed information about classical PHTS-associated phenotypes, such as NDD, making this cohort inaccessible for the NDD versus cancer phenotype comparison as we presented using our Cleveland Clinic PHTS series. It may be tempting to speculate that the lower frequency of individuals with PHTS and germline variants in other NDD-related genes in All of Us participants may be attributed to shortened lifespan, resulting in underestimated prevalence in an adult population. However, this is refuted by our data showing that more than two-thirds of participants from the PTEN Multidisciplinary Clinic series harboring germline variants in NDD-related genes are 18 years of age and older (median [IQR] age at consent, 36 [10–50] years).

Because known cancer and NDD associated gene variants only occurred in a subset of individuals with PHTS and these phenotypes, we then performed an agnostic genome-wide approach to identify candidate genomic modifiers. We acknowledge that the common variant analysis is limited by the modest size of the PHTS cohort, which reduces statistical power and increases susceptibility to deviations from null expectations, as reflected by early divergence in the Q–Q plot. This limitation is inherent to the rarity of PHTS. Nevertheless, to our knowledge, this represents the largest PHTS cohort assembled to date. Importantly, the common variant analysis is intended to be exploratory and hypothesis-generating rather than definitive. Accordingly, results are interpreted cautiously, without causal inference from individual associations, and are presented to prioritize candidate loci and biological pathways for validation in larger, independent cohorts. Through the rare variant analysis, of the top candidates, we identified *ZNF713*, a transcription factor associated with ASD when a CGG-repeat expansion variant is present^28^. *TPTE2P1*, encoding Transmembrane Phosphoinositide 3-Phosphatase and Tensin Homolog 2 Pseudogene 1, represents a pseudogene of *TPTE2*, a gene which is homologous to *PTEN*^29^. Finally, *PDPK1* encoding 3-phosphoinositide dependent protein kinase 1 enables 3-phosphoinositide-dependent protein kinase activity, thus regulating PI3K signaling by activating kinases of the AGC family, including AKT^30^. Intriguingly, these genes may contribute to tumorigenic and/or neurodevelopmental phenotypes, suggesting that their cross-talk with the germline background of impaired PTEN function may contribute to shaping the eventual manifested outcomes.

Taken together, our findings underscore the importance and complexity of genomic modifiers in shaping the phenotypic spectrum of PHTS. By delineating the modifier loci and molecular pathways that interact with PTEN dysfunction, future work will refine our understanding of disease heterogeneity, illuminate mechanisms of oncogenesis and developmental dysregulation, and establish a foundation for broader insights into gene-environment and gene-gene interactions in cancer susceptibility syndromes.

## Methods

### Research participants

We scanned our clinical relational database for accrued research participants with archived DNA. Research participants considered were accrued from September 1, 2005, through December 1, 2020. We obtained Cleveland Clinic Institutional Review Board approval (protocols 8458 and 15–174) and informed written consents from all research participants. The study was conducted in agreement with the principles of the Declaration of Helsinki. Participants were evaluated at the *PTEN* Multidisciplinary Clinic and Center of Excellence at the Cleveland Clinic (Cleveland, Ohio, USA), or at a Developmental Synaptopathies Consortium site (Boston Children’s, Cincinnati Children’s, Stanford University, or University of California, Los Angeles). We prioritized participants with confirmed germline *PTEN* testing results and regardless of specific phenotypes. Baseline information including any cancer and/or neurodevelopmental disorder (NDD) history was recorded at the time of consent. NDD includes autism spectrum disorder (ASD), developmental delay, and/or intellectual disability based on Diagnostic and Statistical Manual of Mental Disorders (DSM) criteria. Checklists to document specific clinical features were completed by specialist genetic counselors or physicians. Specialist genetics staff reviewed all checklists and if necessary, corresponded with the enrolling center to obtain primary documentation of medical records and pathology reports for phenotypic confirmation with patient consent. We reviewed medical records for each research participant and extracted family history from clinical notes associated with cancer genetics and/or genetic-counseling visits, where applicable and with the participants’ consent.

### Germline *PTEN* variant classification

Participants in this study included probands and a subset of family members who have germline *PTEN* variants that are predicted to be pathogenic and/or likely pathogenic (P/LP, *n* = 481), variants of uncertain significance (VUS, *n* = 79), variants with conflicting interpretation of pathogenicity (at least one classification should be a P, LP, and/or VUS, *n* = 11), and variants predicted to be likely benign (*n* = 8). We included *PTEN* variants classified as likely benign and VUS because participant carriers displayed various clinical presentations of PHTS, including thyroid nodules, macrocephaly, gastrointestinal polyps, skin tags, lipomas, Hashimoto’s disease, and others, with or without NDD features and/or cancer. We classified *PTEN* variants into tier 1, including all pathogenic and/or likely pathogenic variants, and tier 2, including all other variants. *PTEN* variant classifications were ascertained by clinical genetic testing reports where available, ClinVar database classifications, and/or the ClinGen gene-specific criteria for *PTEN* variant curation^31^.

### Whole genome sequencing and alignment

We subjected germline genomic DNA extracted from peripheral-blood leukocytes of the eligible research participants to genome sequencing. We performed PCR-free whole-genome sequencing (WGS) with a target mean raw coverage of 30x. Sequencing was performed at the Broad Institute Clinical Research Sequencing Platform (Cambridge, Massachusetts, USA). Raw sequencing reads were aligned to the human reference genome (hg38) using the Burrows-Wheeler Aligner (BWA, v0.7.17)^32^. Post-alignment processing, including base quality score recalibration and duplicate read removal, was performed with the Genome Analysis Toolkit (GATK, v4.3)^33^, following GATK Best Practices for raw read alignment^34^. Germline single nucleotide polymorphisms (SNPs) and insertions/deletions (INDELs) were identified using GATK HaplotypeCaller, employing local haplotype reassembly as recommended in best practices^34^. Identified variants were annotated with the Ensembl Variant Effect Predictor (VEP)^35^, and annotated VCF files were subsequently converted to Mutation Annotation Format (MAF) using vcf2maf (v1.6.21)^36^. To predict clinically relevant functional impact, variants were annotated using ANNOVAR (with ClinVar version 20220320).

### Evaluation of genes known to be associated with cancer and NDD

Cancer-related genes covered 85 genes typically tested clinically in multi-gene panels (Supplementary Data 1), including actionable cancer associated genes as defined by the American College of Medical Genetics and Genomics (ACMG) policy statement^37^. Genes associated with NDD covered three independent clinically tested panels (Ambry NeurodevelopmentNext™ [202 genes]; Ambry AutismNext® [72 genes]; Invitae Neurodevelopmental Disorders (NDD) Panel [241 genes]; last accessed March 11, 2024), whose overlap results in 331 unique genes (Supplementary Data 2). Bed files were generated for each gene list and regions extracted from the genome sequences. Following variant filtration and quality control, we retained variants predicted to be pathogenic and/or likely pathogenic (P/LP) according to ClinVar classifications. For unreported variants, Franklin by Genoox (RRID:SCR_024547; last accessed September 10, 2025) was used along with expert opinion at the Cleveland Clinic PTEN Multidisciplinary Clinic and Center of Excellence. Expert opinion consensus involved five tier 1 (frameshift truncating, nonsense, and splicing) germline variants and two independent evaluators (L.Y. and Y.N.). We excluded heterozygous variants in genes associated with autosomal recessive disorders, implying carrier status. All variants were inspected manually using the Integrative Genomics Viewer (IGV)^38^.

### Analysis of the All of Us dataset

We analyzed the controlled tier dataset (version 8) of the All of Us (AoU) research program to investigate the prevalence of genes known to be associated with cancer and NDD in an unselected population-based series of participants with PHTS. This is in contrast with individuals from a specialized clinical cohort, as reflected in the Cleveland Clinic’s PTEN Multidisciplinary Clinic series. At the cut-off date of October 1, 2023, the program has enrolled 633,547 participants, out of which 353,834 have phenotypic information and 414,830 have short-read whole genome sequencing (srWGS) data. Of those, 55 participants were identified to have pathogenic and/or likely pathogenic (P/LP) *PTEN* variants. We implemented an identical protocol to extract and annotate variants in known cancer and NDD associated genes, as was performed for our participants from the Cleveland Clinic PTEN Multidisciplinary Clinic.

### Genomic burden testing

We performed genotype-level quality control on genome-wide variants, sample-level quality control, ancestral stratification, and genetic relatedness analysis. We filtered single-nucleotide polymorphisms (SNPs) with low genotyping rates (<10%), samples with missingness (<10%), and deviation from Hardy-Weinberg equilibrium (*P* ≤ 10^−50^). This resulted in 563 samples passing all quality control metrics. After excluding *PTEN* wildtype family members for the primary analyses, the analytic sample consisted of 543 probands with germline *PTEN* variants. We performed two main analyses using the Generalized Mixed Model Association Test (GMMAT) analysis^16^. The single common variants (minor allele frequency or MAF between 0.01 and 0.5), score test (glmm.score) was performed with additional Wald test to estimate the effect size for top significant variants. For rare coding and non-coding variants (MAF≤0.01), the variant Set Mixed Model Association Tests (SMMAT) was performed, which include the burden test, the sequence kernel association test (SKAT), SKAT-O and an efficient hybrid test of the burden test and SKAT. GMMAT controls for genetic ancestry and population structure through a linear mixed-model framework that explicitly models genome-wide genetic relatedness among individuals. GMMAT incorporates a genetic relationship matrix (GRM) constructed from genome-wide variants. By including the GRM as a random effect, GMMAT effectively adjusts for ancestry-driven correlation in phenotypes, which is particularly well-suited for unbalanced case-control designs and small cohorts. The genome-wide gene range list was obtained from plink resource (glist-hg38: https://www.cog-genomics.org/plink/1.9/resources, based on UCSC genome browser). Age at consent, biological sex, and *PTEN* variant tier were used as covariates in the GMMAT model. Additionally, we included the genomic relatedness matrix as a covariate in the model, which captures the genetic similarity between individuals to model relatedness and shared genetics. The gene-level effect size beta was calculated from burden test score and variance, as B.score/B.var, and the standard error (SE) of beta was calculated as 1/sqrt(B.var), where B refers to the burden test.

### Statistical analysis

Clinical and demographic characteristics among participants are described and comparisons tested using a Chi-square test for categorical variables or *t*-test for continuous variables. Analyses were performed using R software version 4.2.1 (R Project for Statistical Computing) and OpenEpi version 3.01 (Open Source Epidemiologic Statistics for Public Health) software. All statistical tests were two-sided, and *P* < 0.05 were deemed significant. We performed multiple testing correction via False Discovery Rate (FDR) estimation.

## Supplementary information

Supplementary Information

Supplementary Data 1

Supplementary Data 2

Supplementary Data 3

Supplementary Data 4

Supplementary Data 5

## Data Availability

The data generated in this study will be publicly available in the database of Genotypes and Phenotypes (dbGaP) upon acceptance. All of Us (AoU) data analyses were performed using the AoU Controlled Tier Dataset v8 dataset and executed using the AoU Research Workbench. All other raw data are available from the corresponding author on reasonable request.

## Code availability

All software versions are indicated within the Methods. All custom scripts related to this study are available from the corresponding author upon reasonable request. All other bioinformatics and statistical tools are publicly available and are described within the manuscript text.
