# Supplementary material for: Genomic modifiers of malignant and neurodevelopmental phenotypes in individuals with PTEN hamartoma tumor syndrome
Source: NPJ Genom Med. 2026 Mar 17;11:25. doi: 10.1038/s41525-026-00556-1 (PMC13172402; doi:10.1038/s41525-026-00556-1)
Supplement: Supplementary file 1 — Supplementary Information [file 41525_2026_556_MOESM1_ESM.pdf]

Supplementary Data 1: List of studied cancer-related genes

Cancer-related genes covered 85 genes typically tested clinically in multi-gene panels, including actionable genes as defined by the American College of Medical Genetics and Genomics (ACMG).

Supplementary Data 2: List of studied NDD/ASD-related genes

Genes associated with NDD/ASD covered three independent clinically tested panels including Ambry NeurodevelopmentNext™ (202 genes), Ambry AutismNext® (72 genes]), and Invitae Neurodevelopmental Disorders (NDD) Panel (241 genes), whose overlap results in 331 unique genes.

Supplementary Data 3: Genotype and phenotype characteristics of carriers of cancer-related gene variants

We identified 37 individuals with PHTS and germline variants in other cancer-related genes. The table includes the identified variants, pathogenicity classifications, *PTEN* variant tier, as well as demographic and cancer-specific phenotypes of carriers.

Abbreviations: CCF, Cleveland Clinic Foundation; F, female; M, male; ca, cancer; BCC, basal cell carcinoma; GU, genitourinary; NOS, not otherwise specified.

Supplementary Data 4: Genotype and phenotype characteristics of carriers of NDD/ASD-related gene variants

We identified 43 individuals with germline variants in NDD/ASD-associated genes. The table includes the identified variants, pathogenicity classifications, *PTEN* variant tier, as well as NDD/ASD-associated phenotypes of carriers.

Abbreviations: CCF, Cleveland Clinic Foundation; F, female; M, male.

Supplementary Data 5: List of genome-wide common variants

Genome-wide significant common variants (MAF > 0.01) included 622,149 common variants with *P* value <0.05, of which 748 variants have a *P* value < $5x10^{-8}$.

Abbreviations: SNP, single nucleotide polymorphism; POS, position; A1, allele 1; A2, allele 2; N, number; AF, allele frequency; VAR, variance; SE, standard error; PVAL, P value.
